# TNFR1, TNFR2, neutrophil gelatinase-associated lipocalin and heparin binding protein in identifying sepsis and predicting outcome in an intensive care cohort

**DOI:** 10.1038/s41598-020-72003-9

**Published:** 2020-09-18

**Authors:** Maria Bergquist, Line Samuelsson, Anders Larsson, Jonas Tydén, Joakim Johansson, Miklos Lipcsey

**Affiliations:** 1 Department of Medical Sciences, Clinical Physiology, Uppsala University, Uppsala, Sweden; 2 Department of Surgical and Perioperative Sciences, Anaesthesiology and Critical Care Medicine (Östersund), Umeå University, Umeå, Sweden; 3 Department of Medical Sciences, Clinical Chemistry, Uppsala University Hospital, Uppsala, Sweden; 4 Hedenstierna Laboratory, CIRRUS, Anaesthesiology and Intensive Care, Department of Surgical Sciences, Anesthesiology, Uppsala University, Uppsala, Sweden

**Keywords:** Biomarkers, Medical research, Molecular medicine

## Abstract

To date no biomarkers can aid diagnosing sepsis with adequate accuracy. We set out to assess the ability of Tumor necrosis factor receptor (TNFR) 1 and 2, Neutrophil gelatinase-associated lipocalin (NGAL) and Heparin binding protein (HBP) to discriminate sepsis from non-infected critically ill patients in a large ICU cohort, and to evaluate their value to predict mortality at 30 days. Adult patients admitted to the ICU with an arterial catheter were included. Clinical data and blood samples were prospectively recorded daily. Diagnoses were set retrospectively. Descriptive statistics and logistic regression models were used. NGAL, TNFR1 and TNFR2 were higher in sepsis patients compared to other diagnoses, as well as in non-survivors compared to survivors. In addition, these biomarkers increased with increasing stages of acute kidney injury. TNFR1 and TNFR2 performed similarly to NGAL and CRP in identifying sepsis patients, but they performed better than CRP in predicting 30-day mortality in this ICU cohort. Thus, TNFR1 and TNFR2 may be particularly useful in identifying high risk sepsis patients and facilitate relevant health care actions in this group of sepsis patients.

## Introduction

Initiating treatment for sepsis has major impact on outcome^1,2^. However, differentiating sepsis from other causes of critical illness can be challenging. Most biomarkers for diagnosing severe infections measure inflammatory activity that are also triggered by other conditions with systemic inflammation such as burn injuries or after major surgery^3,4^. In the intensive care unit (ICU) where patients with systemic inflammation are common, biomarkers with high specificity for sepsis are of great interest. As the cellular response of the innate immune system to microbial triggers is an early and key event in the inflammatory response of sepsis^5^ biomarkers related to these events could be useful for identifying patients with sepsis.

Tumor necrosis factor (TNF) is a potent pro-inflammatory cytokine with a pivotal signaling role in the host defense against infection and injury, as well as controlling the survival of target cells^6^. It exerts immunological effects through two cell surface receptors, TNFR1 (p55TNFR) and TNFR2 (p75TNFR). TNFR1 is the main mediator of TNF and ubiquitously expressed while TNFR2 is mainly expressed on immune cells^7^. Activation through ligand binding of TNFR1 activates mitogen-activated protein kinase pathways and IkB kinase complex, to initiate apoptosis^8^. In contrast, activation of TNFR2 will mobilize and activate NFkB, a transcription factor complex promoting cell survival and proliferation^6^. Although TNFRs are initially membrane-anchored proteins, previous studies have reported proteolytic shedding of TNF receptors during inflammation and after TNF stimulation^9–12^. TNFR1 shedding has also been observed to precede the secretion of TNF in vitro and in vivo^13^, suggesting that increased circulating levels of TNFR1 may function to neutralize the effects of TNF to dampen an exaggerated host immune response. An increased concentration of circulating TNFRs in combination with a decreased concentration of available cell surface receptor proteins, could result in a significant attenuation of the pro-inflammatory TNF activity and bioavailability. Supporting this hypothesis, defective shedding has been observed to cause innate immune hyperresponsiveness and in vivo toxicity from TNF and LPS^13^. To our knowledge, although TNFRs have been suggested as potential biomarkers of sepsis, they have not previously been evaluated in discriminating sepsis from other inflammatory conditions found in the ICU setting.

Neutrophil gelatinase-associated lipocalin (NGAL) is a critical component of innate immunity to bacterial infection^14^, released by neutrophils and macrophages as well as renal tubular cells^15^, explaining why it is best known as a marker of acute kidney injury (AKI)^15^. It is considered a very early predictor of renal dysfunction as its elevation can be detected 24–36 h prior to changes in serum creatinine. In addition to AKI, NGAL has also been observed to be elevated in coronary artery disease and heart failure, and to be a strong predictor of outcome^16,17^. In recent studies, NGAL concentrations have been associated with inflammatory response, endothelial activation and clinical outcome in sepsis^18–20^. However, it has not been confirmed if increased concentrations of plasma NGAL could be used as an early biomarker to identify patients with sepsis from patients with inflammation rising from other causes than infection.

In several severe inflammatory conditions seen in the ICU setting, vascular leakage constitutes a major clinical challenge^21^ and is thought to be driven by proteins released by granulocytes. Heparin Binding Protein (HBP) is released immediately from neutrophil vesicles upon neutrophil activation and besides having anti-microbial and chemotactic characteristics, has also been observed to increase vascular permeability^22^. As both capillary leakage and neutrophil activation are highly dysregulated in patients with sepsis, it is possible that HBP could be detected early during infection and be predictive of outcome and mortality in sepsis. Previous studies have associated HBP levels to circulatory, respiratory and renal failure, as well as to mortality^23–27^. If HBP can be used in discriminating infectious conditions from non-infections inflammation in the ICU remains to be confirmed. Our hypothesis was that TNFR1 and 2, NGAL and HBP could identify patients with sepsis in the ICU.

The objective of this study was to assess the ability of these biomarkers to discriminate sepsis from non-infected critically ill patients in a large ICU cohort, and to evaluate their value to predict mortality at 30 days in this mixed ICU population.

## Results

**Figure 1:**
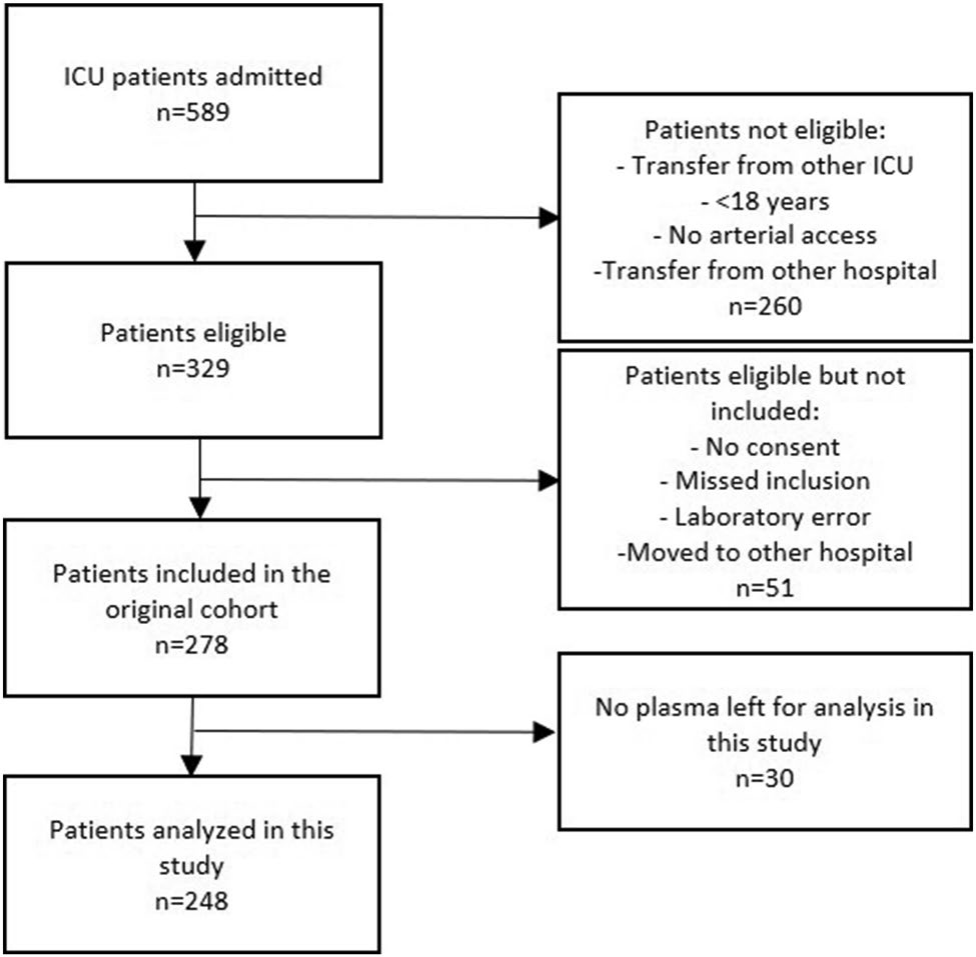
Flow of patients.

Out of 589 patients admitted to the intensive care unit, the numbers of eligible and included patients and reasons for exclusions are presented in Fig. 1. Demographics of the cohort and the subgroups of patients with sepsis and no sepsis are presented in Table 1, and of patients with medical events with low inflammation, trauma and other medical conditions in Suppl Table 1. Patient age, SAPS3 score, SOFA score and 30 day mortality was relatively lower in the group of patients with trauma compared to the other diagnosis groups. Mortality at 30 days was highest in the other medical conditions diagnosis group (33%).

**Table 1:** Patient demographics and clinical parameters.

	All patients (n = 278)	Sepsis (n = 89, 32%)	Non-sepsis (n = 189, 68%)
Age, years	68 (53–76)	73 (62–76)	67 (49–75)
Female sex, n (%)	106 (38)	35 (39)	71 (38)
SAPS3 score	58 (48–70)	65 (56–74)	54 (46–66)
Max SOFA	6 (4–10)	8 (6–11)	4 (2–7)
ICU LOS, days	2 (1–3)	3 (1–6)	2 (1–3)
Mortality at 30 days, n (%)	64 (23)	18 (20)	46 (24)
AKI, n (%)	99 (35)	44 (49)	55 (29)
ARDS, n (%)	33 (12)	16 (18)	13 (7)
**Bacterial foci, n (%)**			
Respiratory		30 (34)	
Bacteremia with unknown focus		18 (20)	
Abdominal		16 (18)	
Urogenital		10 (11)	
Wound/Soft tissue		8 (9)	
CNS		6 (7)	
Endocarditis		1 (1)	

Data are presented as median (IQR) unless otherwise stated.

*SAPS* simplified acute physiology score, *SOFA* sequential organ failure assessment, *ICU* intensive care unit, *LOS* length of stay, *AKI* acute kidney injury, *ARDS* acute respiratory distress syndrome, *CNS* central nervous system.

### Biomarker concentrations in critically ill patients

**Figure 2:**
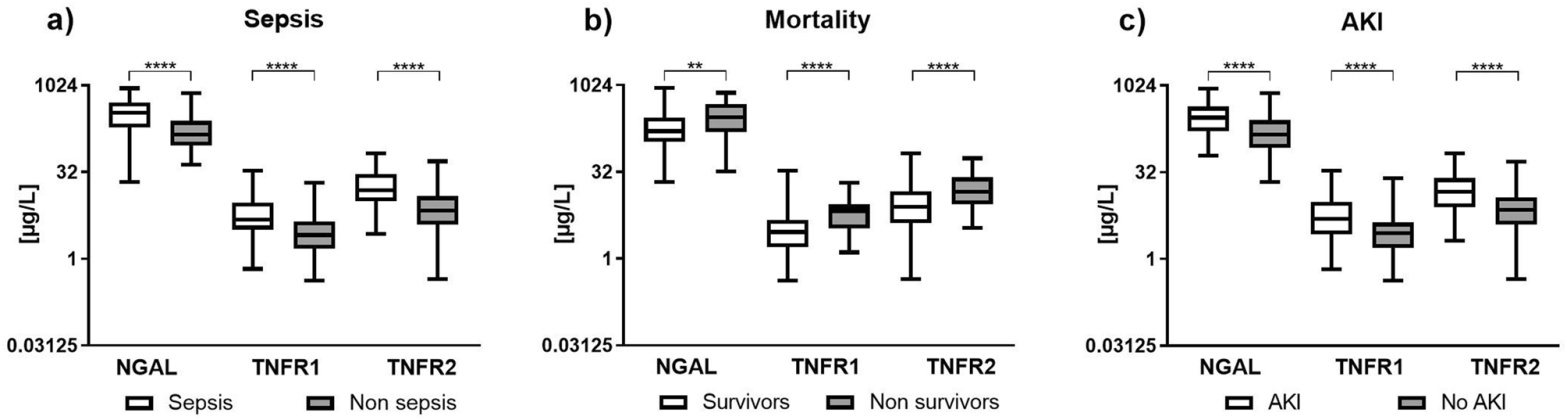
Biomarker concentrations in plasma from patients on admission to the ICU stratified by diagnostic groups; sepsis (n = 89) and non-sepsis (n = 189), survivors (n = 214) and non-survivors (n = 64), and presence of acute kidney injury (AKI, n = 99) and no AKI (n = 179). Box plot summaries of NGAL, TNFR1 and TNFR2 concentration are displayed in natural logarithm scale. Asterisks indicate statistical difference between groups using Mann–Whitney U test. **p < 0.01, ****p < 0.0001.

**Figure 3:**
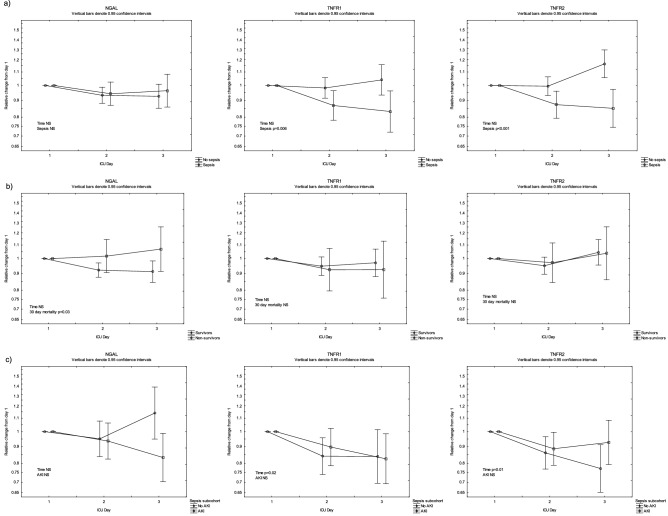
Plasma concentrations NGAL, TNFR1 and TNFR2 from admission (Day 1) to Day 3 in the ICU, stratified by (**a**) sepsis (n = 89) and non-sepsis (n = 189), (**b**) survivors (n = 214) and non-survivors (n = 64), and presence of acute kidney injury (AKI, n = 99) and no AKI (n = 179). ANOVA III for repeated measures was used to assess differences over time.

The concentrations of plasma NGAL, TNFR1 and TNFR2 in patients with sepsis and non-sepsis on admission are illustrated in Fig. 2a. HBP levels have been reported previously^24^. Biomarker concentrations in the subgroups trauma, medical events with low inflammation and other medical conditions are displayed in Suppl Fig. 1. The highest mean concentrations of these biomarkers were all found in the sepsis group, and this difference was consistent over the three ICU days for TNFR1 and TNFR2 (Fig. 3a).

### The performance of biomarkers at cut-off values

The c-indexes for all biomarkers were > 0.7 for NGAL, TNFR1 and TNFR2 for discriminating patients with sepsis from patients without sepsis (Table 2). In comparing sepsis with all other diagnosis groups, CRP displayed the highest c-index and WBC the lowest. For discriminating sepsis from non-sepsis patients at the identified best cut-off, HBP had highest sensitivity, whereas CRP had the highest specificity, negative predictive value (NPV) and positive predictive value (PPV). For discriminating sepsis vs. medical events with low inflammation and trauma patients at the identified best cut-off, CRP had highest sensitivity, specificity, NPV, and PPV, although TNFR2 demonstrated equally high sensitivity in discriminating sepsis from trauma patients. Combining biomarkers added little improvement to the models in identifying sepsis patients.

**Table 2:** Performance of biomarkers in discriminating sepsis patients from non-sepsis patients as well as survival.

Biomarker levels on admission	C-index	Best cut-off	Sensitivity	Specificity	PPV	NPV
**Sepsis vs non-sepsis patients**						
HBP (µg/L)	0.67 (0.60–0.74)	30	87	43	41	87
NGAL(pg/L)	0.77 (0.71–0.83)	185	79	69	56	87
TNFR1 (ng/L)	0.74 (0.67–0.81)	3.0	81	57	48	86
TNFR2 (ng/L)	0.78 (0.72–0.84)	9.0	81	65	54	87
WBC (× 10^9^)	0.41 (0.34–0.48)	–	–	–	–	–
CRP (mg/L)	0.83 (0.77–0.89)	44	86	73	62	91
All biomarkers without WBC	0.86 (0.81–0.91)		76	86		
**Sepsis vs trauma**						
HBP (µg/L)	0.76 (0.66–0.86)	40	63	74	85	44
NGAL (pg/L)	0.86 (0.78.0.94)	185	79	84	94	58
TNFR1 (ng/L)	0.86 (0.78–0.94)	3.4	74	80	92	51
TNFR2 (ng/L)	0.89 (0.82–0.96)	6.4	90	72	90	72
WBC (× 10^9^)	0.46 (0.35–0.57)	–	–	–	–	–
CRP (mg/L)	0.90 (0.83–0.97)	27	90	87	95	74
All biomarkers without WBC	0.95 (0.90–0.99)		93	86		
**Sepsis vs medical events with low inflammation**						
HBP (µg/L)	0.71 (0.63–0.79)	30	84	55	69	75
NGAL(pg/L)	0.81 (0.74–0.88)	175	80	77	81	77
TNFR1 (ng/L)	0.77 (0.70–0.84)	3.0	81	62	72	73
TNFR2 (ng/L)	0.82 (0.75–0.89)	8.7	80	69	72	78
WBC (× 10^9^)	0.44 (0.35–0.53)	–	–	–	–	–
CRP (mg/L)	0.88 (0.82–0.94)	44	86	78	84	81
All biomarkers without WBC	0.92 (0.87–0.97)		82	95		
**30 day mortality**						
HBP (µg/L)	0.64 (0.56–0.72)	39	69	59	33	86
NGAL(pg/L)	0.65 (0.57–0.73)	248	58	69	33	86
TNFR1 (ng/L)	0.73 (0.65–0.81)	4.8	64	75	40	89
TNFR2 (ng/L)	0.71 (0.63–0.79)	12	67	67	34	89
WBC (× 10^9^)	0.59 (0.51–0.67)	12.2	64	54	31	83
CRP (mg/L)	0.57 (0.49–0.65)	39	65	55	30	84
All biomarkers	0.72 (0.64–0.80)		84	50		

Logistic regression model on biomarkers at admission to intensive care.

*HBP* heparin binding protein, *NGAL* neutrophil gelatinase-associated lipocalin, *TNFR* tumor necrosis factor receptor, *WBC* white blood cell count, *CRP* C-reactive protein.

When adjusting for age, SOFA score on admission, SAPS3, and ICU LOS, only HBP and WBC were not independent predictors of sepsis when compared to patients in the medical events with low inflammation group.

### Biomarker association with renal function

**Figure 4:**
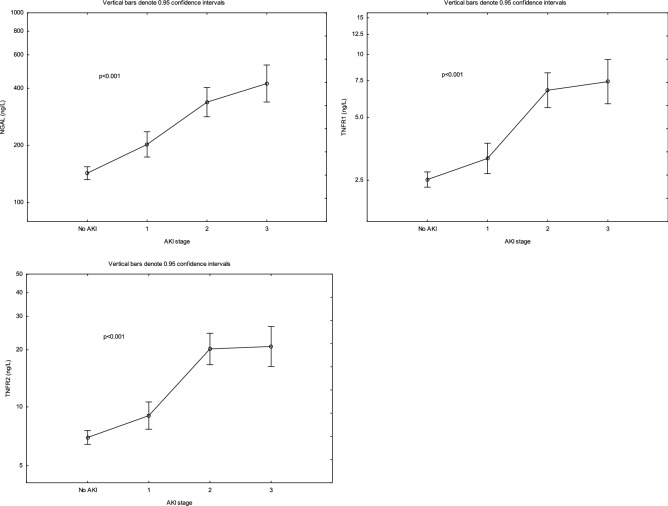
Plasma concentrations NGAL, TNFR1 and TNFR2 in patients stratified by AKI stage. ANOVA III for repeated measures was used to assess group differences.

Plasma concentrations of NGAL, TNFR1 and TNFR2 were higher in patients with AKI compared to patients without AKI (Fig. 2c) and did not change over the three ICU days (Fig. 3b). When stratifying patients according to their AKI stage, NGAL, TNFR1 and TNFR2 increased with increasing level of AKI (Fig. 4).

### Biomarker association with outcome

Plasma concentrations of NGAL, TNFR1 and TNFR2 were higher in the non-survivors as compared to survivors as measured on admission to the ICU (Fig. 2b). While TNFR1 and TNFR2 concentrations did not change significantly during the three ICU days, the NGAL concentrations diverged between survivors and non-survivors (Fig. 3c).

The c-indexes for all biomarkers were > 0.6 for NGAL, TNFR1 and TNFR2 for discriminating survivors from non-survivors (Table 2) with the lowest c-index value for WBC and the highest for TNF1 and TNFR2. For discriminating survivors from non-survivors at the identified best cut-off, HBP had highest sensitivity, TNFR1 the highest specificity. PPV were low for all four biomarkers for mortality at 30 days. NPV were high for all biomarkers with the highest values seen for TNFR1 and TNFR2.

In the univariate regression analysis (Table 3) all biomarkers exhibited significant correlations to age, SOFA score, SAPS3, and ICU LOS apart from HBP vs ICU LOS. NGAL, TNFR1 and TNFR2 vs SOFA score and SAPS3 showed the highest correlation coefficients.

**Table 3:** Biomarker correlations to age and clinical parameters in all patients at admission.

Biomarker	Age (years)	SOFA score	SAPS3	ICU LOS (DAYS)
HBP	rho = 0.21; p = 0.0006	rho = 0.32; p < 0.0001	rho = 0.32; p < 0.0001	NS
NGAL	rho = 0.20; p = 0.004	rho = 0.50; p < 0.0001	rho = 0.38; p < 0.0001	rho = 0.17; p = 0.01
TNFr1	rho = 0.27; p < 0.0001	rho = 0.49; p < 0.0001	rho = 0.44; p < 0.0001	rho = 0.18; p = 0.008
TNFr2	rho = 0.31; p < 0.0001	rho = 0.52; p < 0.0001	rho = 0.47; p < 0.0001	rho = 0.20; p = 0.003
WBC	NS	NS	NS	NS
CRP	rho = 0.15; p = 0.02	rho = 0.34; p < 0.0001	rho = 0.36; p < 0.0001	rho = 0.22; p = 0.0009

Biomarker correlations to age and clinical parameters were calculated using Spearman’s correlation test in patients at admission to intensive care.

*HBP* heparin binding protein, *NGAL* neutrophil gelatinase-associated lipocalin, *TNFR* tumor necrosis factor receptor, *WBC* white blood cell count, *CRP* C-reactive protein, *SOFA* sequential organ failure assessment, *SAPS* simplified acute physiology score, *ICU* Intensive care unit, *LOS* length of stay.

P < 0.05 was considered statistically significant.

WBC was the only independent predictor of death at 30 days after adjusting for age, SOFA score, SAPS3, and ICU LOS (Table 4).

**Table 4:** Odds rations for increases in biomarkers in patients with sepsis vs trauma, sepsis vs other medical conditions, as well as for patients being alive at 30 days after ICU admission, adjusted for sex, SAPS3, maximal ICU SOFA score and age.

	Odds ratio	95% confidence interval	p-value
**Sepsis vs non-sepsis patients**			
HBP (µg/L)	1.00	(1.00–1.00)	NS
NGAL(pg/L)	1.005	(1.00–1.01)	0.0001
NGAL adjusted for AKI	1.00	(1.00–1.01)	< 0.0001
TNFR1 (ng/L)	1.18	(1.07–1.30)	0.0008
TNFR2 (ng/L)	1.08	(1.04–1.12)	< 0.0001
WBC (× 10^9^)	1.03	(0.99–1.08)	NS
CRP (mg/L)	1.008	(1.00–1.01)	< 0.0001
**Sepsis vs trauma**			
HBP (µg/L)	1.03	(1.00–1.05)	0.03
NGAL(pg/L)	1.01	(1.00–1.02)	0.002
NGAL adjusted for AKI	1.02	(1.01–1.03)	0.003
TNFR1 (ng/L)	1.70	(1.13–2.56)	0.01
TNFR2 (ng/L)	1.20	(1.03–1.38)	0.01
WBC (× 10^9^)	1.00	(0.92–1.07)	NS
CRP (mg/L)	1.02	(1.01–1-03)	0.003
**Sepsis vs medical events with low inflammation**			
HBP (µg/L)	1.00	(1.00–1.01)	NS
NGAL(pg/L)	1.01	(1.00–1.01)	< 0.0001
NGAL adjusted for AKI	1.01	(1.00–1.01)	< 0.0001
TNFR1 (ng/L)	1.29	(1.09–1.52)	0.003
TNFR2 (ng/L)	1.11	(1.04–1.19)	0.002
WBC (× 10^9^)	0.97	(0.92–1.02)	NS
CRP (mg/L)	1.02	(1.01–1.03)	< 0.0001
**30 day mortality**			
HBP (µg/L)	1.00	(1.00–1.00)	NS
NGAL(pg/L)	1.00	(1.00–1.00)	NS
NGAL adjusted for AKI	1.00	(1.00–1.00)	NS
TNFR1 (ng/L)	0.99	(0.93–1.07)	NS
TNFR2 (ng/L)	1.00	(0.97–1.03)	NS
WBC (× 10^9^)	1.06	(1.01–1.11)	0.02
CRP (mg/L)	1.00	(0.99–1.00)	NS

Logistic regression model on biomarkers at admission to intensive care.

*HBP* heparin binding protein, *NGAL* neutrophil gelatinase-associated lipocalin, *TNFR* tumor necrosis factor receptor, *WBC* white blood cell count, *CRP* C-reactive protein, *SOFA* sequential organ failure assessment, *SAPS* simplified acute physiology score, *ICU* Intensive care unit.

*P* < 0.05 was considered statistically significant.

## Discussion

Our data show that HBP, NGAL, TNFR1 and TNFR2 are higher in sepsis patients compared to other diagnoses, suggesting that these biomarkers could be of value in discriminating patients with infection from other severe inflammatory conditions. Of these biomarkers, TNFR2 performed nominally best in discriminating sepsis patients from non-infected medical patients. With the chosen cut-off values for sepsis in this study, CRP had higher sensitivity and specificity than other biomarkers for identifying sepsis patients in this ICU cohort. However, both TNFR1 and TNFR2 performed better than CRP in predicting outcome. WBC was the only independent predictor of mortality at 30 days after adjusting for age and acute and chronic illness severity.

Sepsis is a complex syndrome with systemic inflammation and is still associated with high mortality and its early diagnosis is critical for timely management and patient survival^28^. New clinical and laboratory tools to distinguish between bacterial and non-bacterial conditions are urgently needed to identify patients with severe infections early. Today, there is no available test to diagnose sepsis accurately. Instead, a number of non-specific clinical parameters and the suspicion of infection define sepsis. To confirm suspected infection, several biomarkers are used clinically. The most widely used biomarker is procalcitonin, which has been shown to have some advantages over CRP. Yet procalcitonin has limitations for diagnosis of sepsis and studies on guiding antibiotic therapy with this biomarker have been conflicting regarding patient benefit^29,30^. In the present study we analyzed clinical biomarkers in plasma from a mixed ICU population and found that NGAL and TNFR1 and 2 are associated with poor prognosis and can be used as support in identifying patients with sepsis, especially those with high mortality risk, upon admission to the ICU.

In line with our results, NGAL has previously been associated with increased clinical severity and diagnosis of sepsis^19,31,32^. As NGAL is considered an endogenous bacteriostatic protein expressed to large extent by neutrophils and macrophages, it plays a critical role in the innate immunity against bacterial infection^14^. In contrast to this, it could also hold anti-inflammatory properties by decreasing apoptosis in host immune cells^33^. Taken this into account, it is not surprising that NGAL would increase during sepsis and be of value for the early detection and monitoring of infection. However, identifying patients with sepsis in the ICU is challenging^3^ since activation of an immune response and inflammation is common both with and without infectious etiology^29^. In this population, as shown in the current study, NGAL and CRP are particularly useful for identifying sepsis patients with high specificity and initiating adequate treatment.

Although increased plasma levels of TNFRs in systemic inflammatory conditions have been described decades ago^34^, these soluble receptors of proximal inflammation mediators have not been evaluated as sepsis markers. Although it is a common hypothesis that the shedding of TNF receptors binds and neutralize circulating TNF^34^, it cannot be excluded that TNF receptors act as a reservoir for TNF and thus prolonging its pro-inflammatory action^35^. It is thus not clear if the observed increased shedding of TNFRs in sepsis contribute to a worse outcome or is reflecting an anti-inflammatory response to protect from a deleterious immune reaction to infection. In our study these biomarkers performed with the highest overall combination of sensitivity and specificity for sepsis and were predictors of sepsis even when adjusted for illness severity, age and organ failure. Thus, TNFR1 and 2 could be useful in identifying sepsis patients. Our study does not aim to explain the causal relationship between the increased concentrations of circulating TNFRs and sepsis diagnosis.

Increased HBP levels in sepsis patients in this cohort have been reported previously^24^ and have been investigated as sepsis biomarkers in other critically ill patient populations^36^. Compared to the latter study, HBP had higher c-index in this study for discriminating sepsis patients from other groups. At the set best cut-off, sensitivity was high, negative predictive value was good among the tested biomarkers, but the specificity was low. Thus, sepsis is highly unlikely with low levels HBP in the ICU cohort and using HBP as a biomarker could aid withholding antibiotics in patients without infection.

In this study, 35% of the patients developed AKI, and 49% of the patients with sepsis. Our finding that TNFRs correlate with renal function in sepsis is in agreement with previous studies^37–39^. It has been suggested that elevated concentrations of circulating TNFRs could be an effect of impaired renal clearance following organ dysfunction in sepsis^37^. However, the association between increased TNFRs and renal failure remained even in patients with low serum creatinine, suggesting that impaired renal clearance alone cannot explain the increase in soluble TNFRs^39^. In our study, we observed a strong correlation between TNFRs and AKI stage, which to our knowledge has not previously been documented. The finding that concentrations of NGAL, TNFR1 and TNFR2 are higher in patients with AKI compared to patients without AKI, in combination with that these biomarkers are increasing with AKI stage, supports the hypothesis that AKI in this population is driven by inflammation.

The concentrations of these biomarkers were higher in patients who died compared to patients who survived, which is consistent with previous studies^40^. However, in our study we found that they could not be used as independent predictors of mortality after adjusting for illness severity, age and organ failure. This could be explained by the very heterogeneous group of patients with different infecting agents and varying co-morbidities. Moreover, the prediction of mortality with these biomarkers was adjusted for severity of illness, age and the extent of organ failure that together or separately are good or excellent predictors of mortality.

This study, to the best of our knowledge, is the first large prospective investigation of soluble TNFRs in a mixed ICU cohort, comparing sepsis to other non-infectious inflammatory conditions. To facilitate comparison of our data to other studies and clinical practice, we also report the performance of NGAL and HBP which have been investigated previously, as well as two of the most widely used biomarkers CRP and WBC. Moreover, this cohort of patients includes a wide variety of patients admitted to the ICU, which we consider will reflect the performance of these biomarkers more accurately, rather than drawing conclusions from comparisons with healthy controls. As patients were included consecutively, this study could closer reflect how these biomarkers could be utilized in the clinical environment. Finally, the diagnosis of sepsis was made by two independent experts increasing the accuracy of the diagnosis.

Our study has several limitations. Firstly, the analyses were performed post-hoc on an existing material from a previous study. However, the analysis plan was determined prior to commencing this study. Secondly, data on clinical use of antibiotics or immune modulatory drugs (e.g. glucocorticoids) was not available, which may have affected the plasma concentrations of these biomarkers. Apart from studying TNFR1 and 2 in different populations, future studies on guiding antibiotic therapy with TNFRs, NGAL and HBP according to their sensitivity and specificity would be of value. Another limitation is that this study was not designed to collect microbiological data in this ICU cohort. Patients are usually diagnosed with sepsis on clinical grounds, as positive blood cultures in sepsis cohorts are typically around 40%^41^. However, during the three days after ICU admission, potential community acquired infections are likely to have been captured in this study. Future studies could add further information on the value of using NGAL, TNFR1 and TNFR2 in patients with nosocomial sepsis.

These data suggest that TNFR1 and 2 are promising biomarkers of sepsis in the ICU cohort. The novel biomarkers TNFR1 and TNFR2 performed similarly to NGAL and CRP in identifying sepsis patients. In the same group of patients, high NGAL levels are very suggestive of sepsis, while low HBP levels rule out sepsis. Given its sensitivity and specificity profile, HBP is likely to perform better as a sepsis marker in a cohort with lower incidence of sepsis than the cohort investigated in this study. Additionally, TNFR1 performed better than CRP in predicting 30-day mortality and thus can potentially identify high risk patients with sepsis.

## Patients and methods

### Setting

This prospective observational study was performed at the 8-bed mixed ICU of Östersund hospital, Sweden, a 300-bed hospital in Sweden based on material from a previous study^24^.

### Data collection and patient groups

All patients admitted to the ICU between 1st of February 2012 and 31st of January 2013 were screened for eligibility. Inclusion criteria were: admission to the ICU and presence of or need for an arterial catheter to be inserted. Patients under the age of 18 years and those transferred from other ICUs were excluded. Clinical data were recorded prospectively daily. Final diagnosis was set retrospectively by review of the patients notes.

Simplified acute physiology score 3 (SAPS 3)^42^ was recorded at admission and Sequential organ failure assessment score (SOFA)^43^ was recorded on admission and then on ICU day 1 and 2. The last measurement corresponds to a value most often 48–72 h after admission to the ICU, the exceptions being patients that were discharged, alive or dead, before that registration, where the last available registration was used. Sepsis was defined according to the Sepsis-3 criteria^44^ with suspected or verified infection and a SOFA on admission of 2 or more. Patients with Acute kidney injury (AKI) were identified^45^.

We predefined four diagnosis groups to compare the performance of the biomarkers in identifying sepsis patients similarly to a previous study of ours^46^: Sepsis group, according to the definitions above; Trauma group, presumed to have inflammation without infection on admission; Medical events with low inflammation, presumed to be admitted to the ICU without infection; and the remaining patients were designated to the Other medical conditions group.

### Handling of samples

Blood samples were collected on admission to the ICU and on the two following days. Routine chemistry including white blood cell count (WBC) and C-reactive protein (CRP) was performed in the hospital central laboratory and registered on the day of admission.

### HBP analysis

Concentrations of HPB in plasma were measured as described earlier^24^. Briefly, plates were coated with a mouse monoclonal antibody directed against HBP. Intra-, and inter-assay coefficient of variation were both less than 10%.

### NGAL, TNFR1 and TNFR2

Levels of NGAL (DY1757), TNF receptor 1 (DY225) and TNF receptor 2 (DY726) were analyzed using commercially available ELISA kit (R&D Systems, Minneapolis, MN). The assays had a total coefficient of variation (CV) of approximately 6–7% and the samples were analyzed as singletons. Laboratory technicians were blinded in relation to patient data.

### Statistical analysis

Data are presented as median (IQR) or as number of observations (percent of total number of observations). To compare two or more groups, Mann–Whitney U test or Kruskal Wallis with Dunn’s correction were used, respectively. Spearman’s correlation test was used for assessing the association between two variables. We used ANOVA III for repeated measures to assess differences over time. Logistic regression was performed with diagnosis of Sepsis vs. Trauma and Sepsis vs. Other medical conditions, and 30-day mortality as dependent variables with biomarkers as sole predictors to calculate area under the curve of the receiver operating characteristic (c-index). The best cut-off was defined as maximal distance from the diagonal and receiver operating characteristic curve. In the same model after adjusting for age, sex, extent of organ failure (SOFA) and illness severity (SAPS3), we also assessed if the biomarker in the models was an independent predictor of sepsis or death and the magnitude of such an effect expressed as odds ratios. For calculations and figures, STATISTICA software, version 13.2 (StatSoft, Tulsa, OK) and GraphPad Prism 7.0 for Windows (GraphPad Software Inc, La Jolla, CA, USA) were used. p < 0.05 was considered significant where relevant.

### Ethical approval

The study was approved by the Regional Ethical Review Board (EPN) in Linkoping (No. 2018/16–32). Informed consent was obtained from the patient, or next of kin if the patient was unable give consent. All the experiments were performed in accordance with the relevant guidelines and regulations.

STROBE guidelines were followed for reporting.

## Supplementary information

SI Figure 1. Biomarker concentrations in plasma from patients on admission to the ICU stratified by diagnostic groups; sepsis (n=89), trauma (n=35), medical events with low inflammation (MELI, n=74) and other medical conditions (OMC, n=80). Box plots summaries of NGAL, TNFR1 and TNFR2 concentration are displayed in natural logarithm scale. Asterisks indicate statistical difference in comparison to the sepsis group, using Kruskal Wallis test with Dunn’s correction. **p<0.01, ***p<0.001, ****p<0.0001

Supplementary file 2

## Data Availability

The datasets used and/or analyzed during the current study are available from the corresponding author on reasonable request.
