# Supplementary figures and images for: TNFR1, TNFR2, neutrophil gelatinase-associated lipocalin and heparin binding protein in identifying sepsis and predicting outcome in an intensive care cohort
Source: Sci Rep. 2020 Sep 18;10:15350. doi: 10.1038/s41598-020-72003-9 (PMC7501293; doi:10.1038/s41598-020-72003-9)

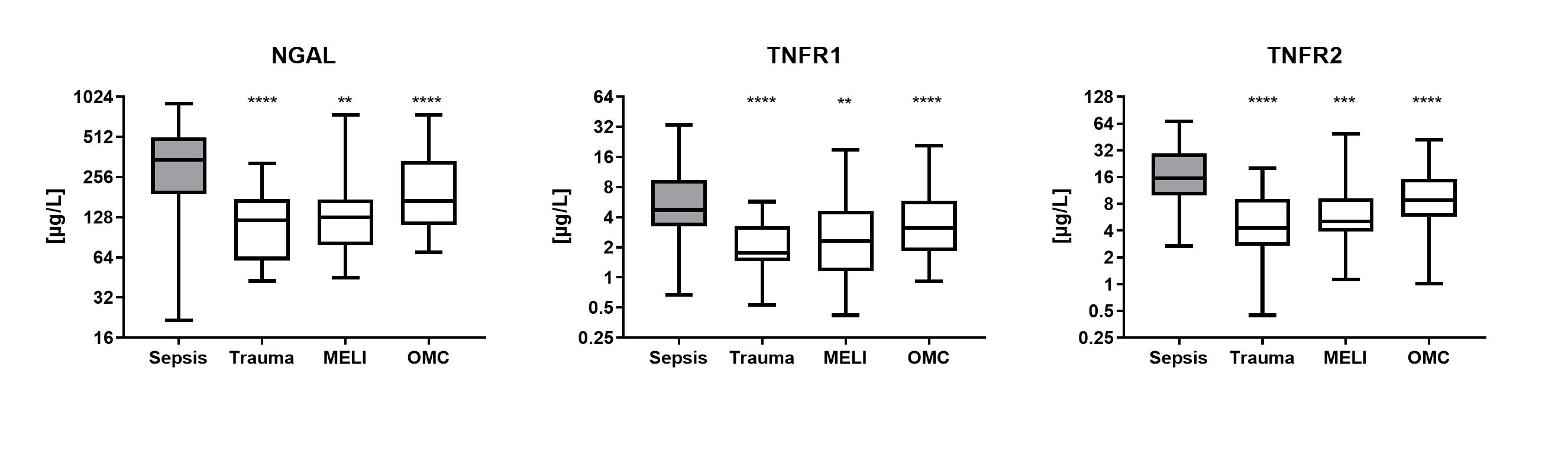

Supplement: Supplementary file 1 — SI Figure 1. Biomarker concentrations in plasma from patients on admission to the ICU stratified by diagnostic groups; sepsis (n=89), trauma (n=35), medical events with low inflammation (MELI, n=74) and other medical conditions (OMC, n=80). Box plots summaries of NGAL, TNFR1 and TNFR2 concentration are displayed in natural logarithm scale. Asterisks indicate statistical difference in comparison to the sepsis group, using Kruskal Wallis test with Dunn’s correction. **p<0.01, ***p<0.001, ****p<0.0001 [file 41598_2020_72003_MOESM1_ESM.jpg]
