# Supplementary material for: TNFR1, TNFR2, neutrophil gelatinase-associated lipocalin and heparin binding protein in identifying sepsis and predicting outcome in an intensive care cohort
Source: Sci Rep. 2020 Sep 18;10:15350. doi: 10.1038/s41598-020-72003-9 (PMC7501293; doi:10.1038/s41598-020-72003-9)
Supplement: Supplementary file 2 [file 41598_2020_72003_MOESM2_ESM.docx]

**Suppl Table 1.** Patient demographics and clinical parameters for non-sepsis patients. Data are presented as median (IQR) unless otherwise stated.

|  | Trauma (n=35, 19%) | Medical events with low inflammation (n=74, 39%) | Other medical conditions (n=80, 42%) |
| --- | --- | --- | --- |
| Age, years | 53 (29 - 72) | 64 (41 - 74) | 71 (64 - 78) |
| Female sex, n (%) | 6 (17) | 36 (49) | 29 (36) |
| SAPS3 score | 47 (39 - 53) | 52 (42 - 62) | 60 (51 - 72) |
| Max SOFA | 4 (3 - 7) | 5 (2 - 7) | 7 (5 - 11) |
| ICU LOS, days | 1 (1 - 3) | 2 (1 - 2) | 2 (1 - 5) |
| Mortality at 30 days, n (%) | 3 (9) | 17 (23) | 29 (36) |
| AKI, n (%) | 8 (23) | 19 (26) | 28 (35) |
| ARDS, n (%) | 3 (9) | 2 (3) | 12 (15) |
| Trauma type |  |  |  |
| Neurotrauma | 15 |  |  |
| Fractures | 15 |  |  |
| Blunt Abdominal trauma | 8 |  |  |
| Blunt thorax trauma | 7 |  |  |
| Common diagnosis |  |  |  |
| Diabetes ketoacidosis |  | 12 |  |
| Intoxication |  | 11 |  |
| Electrolyte disturbance |  | 11 |  |
| Cerebrovascular lesion |  | 8 |  |
| Acute/Chronic Respiratory Failure |  | 5 |  |
| Chronic Cardiovascular Disease |  | 5 |  |
| Seizures |  | 4 |  |
| Anaphylaxis |  | 2 |  |
| Renal Failure |  | 2 |  |
| Other |  | 2 |  |
| Cardiac Arrest |  |  | 16 |
| Pneumonia |  |  | 12 |
| Gastrointestinal Bleeding |  |  | 11 |
| Acute aortic aneurysm |  |  | 9 |
| Pre-eclampsia |  |  | 7 |
| COPD exacerbation |  |  | 6 |
| Gastrointestinal (excl bleed) |  |  | 5 |
| Postoperative care |  |  | 4 |
| Bleeding (excl GI) |  |  | 4 |
| Other |  |  | 2 |

SAPS: simplified acute physiology score, SOFA: sequential organ failure assessment, ICU: Intensive care unit, LOS: length of stay, AKI: acute kidney injury, ARDS: acute respiratory distress syndrome, COPD: Chronic obstructive pulmonary disease.
